# Root cell wall plasticity in iron homeostasis: an overlooked frontier in plant nutrition

**DOI:** 10.1111/nph.70806

**Published:** 2025-12-07

**Authors:** Poonam Kanwar, Petra Bauer

**Affiliations:** ^1^ Institute of Botany Heinrich Heine University D‐40225 Düsseldorf Germany; ^2^ Cluster of Excellence on Plant Science (CEPLAS) Heinrich Heine University D‐40225 Düsseldorf Germany

**Keywords:** apoplast, callose, Casparian strip, cell wall expansion, ROS, suberization

## Abstract

Iron (Fe) is an essential micronutrient for plant growth and development, yet its availability in soils is often limited or excessive, leading to widespread Fe deficiency or toxicity that constrains crop productivity. While Fe uptake, transport, and signaling pathways have been well characterized, the role of the root cell wall as a dynamic regulator of Fe homeostasis remains largely overlooked. This review presents the first comprehensive synthesis of how the structural and biochemical plasticity of the root apoplast and endodermis modulates Fe acquisition and distribution. We highlight key mechanisms, including pectin demethylation, proton extrusion, apoplastic acidification, callose deposition, Casparian strip formation, and suberization, that actively influence Fe solubility, binding, and radial movement across root tissues. By integrating recent findings on root cell‐wall plasticity with Fe regulation, we identify regulatory hubs that link Fe status to cell‐wall remodeling, as well as major knowledge gaps in the signaling pathways that mediate this connection. This timely review introduces a novel perspective that connects physical cell wall dynamics with molecular Fe signaling and underscores the potential of targeting cell wall traits to enhance Fe use efficiency and crop resilience, particularly on marginal soils.

## Introduction

Iron (Fe) is an indispensable micronutrient for plants, essential for photosynthesis, respiration, redox homeostasis, and various enzymatic functions. Plants tightly regulate Fe uptake, distribution, storage, and detoxification to avoid both Fe deficiency, which limits growth and productivity, and Fe toxicity, which leads to reactive oxygen species (ROS) generation and cellular damage (Schwarz & Bauer, 2020). Understanding how this balance is maintained is critical for improving nutrient use efficiency and crop resilience, especially under the mounting pressures of soil degradation and global food insecurity. While molecular mechanisms of Fe homeostasis, such as transporter activity, transcriptional regulation, and systemic signaling, have been extensively characterized, the role of the plant cell wall as a dynamic participant in Fe regulation is only beginning to emerge (Brumbarova *et al*., 2015).

**Fig. 1:**
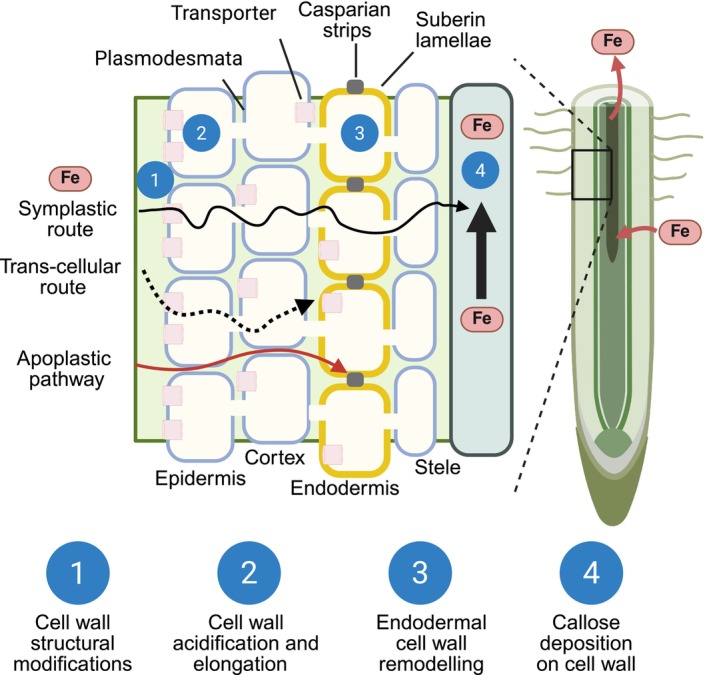
Schematic representation of plant cell wall modifications regulating iron (Fe) uptake, intercellular movement, and cell‐to‐cell transport in roots. The longitudinal section illustrates radial and longitudinal Fe movement through the symplastic (solid black arrows), transcellular (dotted black arrows), and apoplastic (red arrows) pathways. Cell wall remodeling processes, including (1) structural modifications, (2) acidification and elongation, (3) endodermal wall remodeling with Casparian strip formation and suberization, and (4) callose deposition, collectively modulate Fe homeostasis in roots. Figure created with BioRender.com (https://BioRender.com/cu0ex76).

Root tissues, including the epidermis, cortex, and endodermis, regulate the selective transport of water and nutrients via symplastic, apoplastic, and transcellular pathways to the central vasculature (Fig. 1). The root cell wall is a multifunctional interface that governs nutrient sensing, exchange, and signaling. Within the apoplast, negatively charged polysaccharides such as pectins and hemicelluloses bind Fe ions, modulating their mobility and availability (Zhu *et al*., 2016; Peng *et al*., 2021; S. Li *et al*., 2023). Structural modifications, including proton‐driven acidification, pectin demethylation, and cell wall remodeling, actively influence Fe uptake. Diffusion barriers such as the Casparian strip (CS) and suberin layers in the endodermis restrict unregulated Fe entry into the vasculature, while callose deposition under Fe toxicity can limit symplastic connectivity as a defense mechanism (Fig. 1). This review synthesizes emerging research positioning the root cell wall as a central, dynamic hub in Fe homeostasis. We explore key questions, including: How do cell walls remodel in response to Fe status? Which apoplastic components govern Fe retention and mobility? How is Fe movement coordinated across transport pathways? What occurs when endodermal barriers are disrupted? A deeper understanding of these interactions between cell wall remodeling, nutrient regulation, and stress adaptation could guide strategies to improve nutrient management and stress tolerance in crops grown in Fe‐deficient or Fe‐toxic soils.

## Iron and cell wall structural modifications

The first interaction of a nutrient ion with a plant root is adsorption into the ‘apparent free space’ of the apoplast. The root apoplast, primarily formed by the cell wall, is a critical interface between plants and their environment. The cell wall contributes to structural support, cellular shape, tissue protection, and intercellular communication (Vaahtera *et al*., 2019). The primary cell wall, composed of proteins and polysaccharides like cellulose, hemicellulose, and pectin, facilitates the diffusion of water, nutrients, and signaling molecules (Vaahtera *et al*., 2019). The cell wall contains highly negatively charged sites that may serve as a sink for most cationic nutrients. Hemicellulose‐bound iron was found to represent the largest pool of iron in the cell wall (Lei *et al*., 2014). Fe enters roots primarily through the apoplast, where it serves as an essential storage pool (Liu *et al*., 2023). It is estimated that at least 75% of the total Fe in roots is located in the root apoplast (Bienfait *et al*., 1985). The reallocation of apoplastic Fe is crucial for plants under Fe deprivation; however, the regulatory mechanisms behind this process remain unclear.

**Fig. 2:**
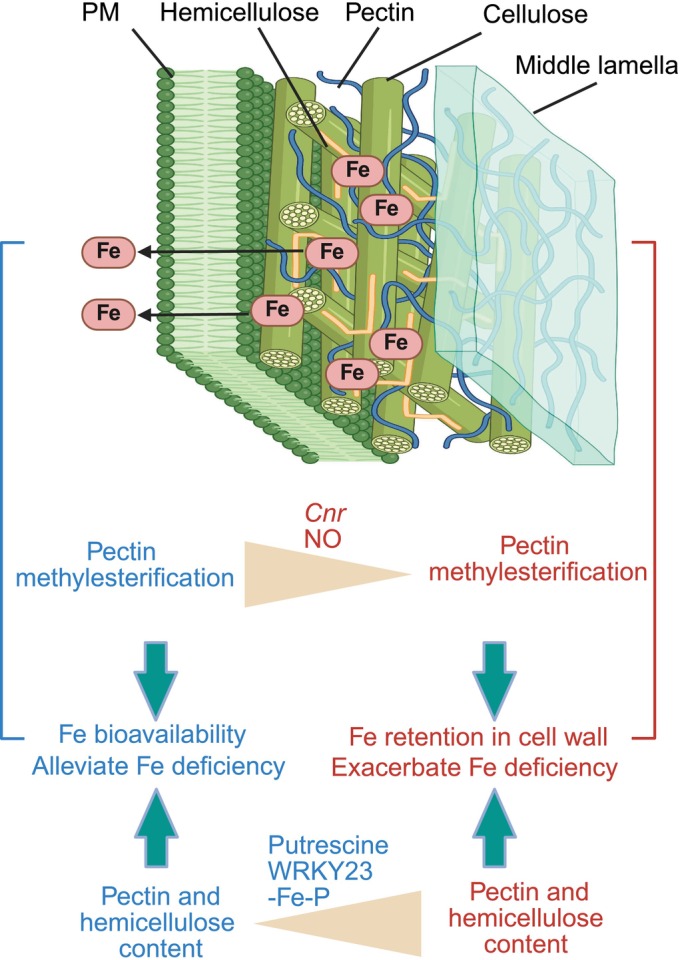
Schematic representation of cell wall modifications regulating iron (Fe) retention and bioavailability in roots. Pectin and hemicellulose in the root cell wall act as major reservoirs for Fe. The degree of pectin methylesterification influences Fe binding: highly methylesterified pectin reduces Fe retention and enhances Fe mobility, whereas demethylesterified pectin increases Fe immobilization, exacerbating deficiency. The abundance of cell wall polymers also modulates Fe retention in the apoplast. Triangles indicate changes in methylesterification or polymer content; arrows denote regulatory relationships. Blue text represents processes promoting Fe bioavailability, and red text indicates those enhancing Fe retention. Cnr, colorless nonripening; NO, nitric oxide; –Fe–P, combined Fe and phosphate deficiency. Figure created with BioRender.com (https://BioRender.com/r7pqi1v).

Structural modifications of the cell wall determine whether Fe is retained within the wall matrix or bioavailable for uptake. Specifically, changes in the methylation status of cell wall polymers, such as pectin and hemicellulose, play a pivotal role (Fig. 2). Elevated activity of pectin methylesterase in mutants such as the tomato *Colorless nonripening* (*Cnr*) epimutant increases pectin demethylation, enhancing Fe retention in the apoplast but exacerbating Fe‐deficiency responses (Zhu *et al*., 2024). Nitric oxide production further modulates Fe bioavailability by decreasing pectin methylation, thereby enhancing the level of Fe stored in the root apoplast in Fe‐deficient roots (Ye *et al*., 2015).

The abundance and composition of cell wall polymers also influence Fe mobilization (Fig. 2). Polyamines such as putrescine alleviate Fe deficiency by facilitating reutilization of apoplastic Fe in Arabidopsis roots by decreasing cell wall hemicellulose contents (Zhu *et al*., 2016). Auxin‐induced expression of the transcription factor *WRKY23* activates *PECTIN LYASE‐LIKE1* (*PLL1*) and *PECTIN LYASE‐LIKE3* (*PLL3*), which promote pectin degradation in the apoplast. This process facilitates Fe mobilization and reutilization in roots when Fe availability is limited (Zhang *et al*., 2025). Under combined Fe and P deficiency (–Fe–P), hemicellulose 1 content and its Fe‐binding capacity decrease, promoting reutilization of root cell wall Fe and enhancing shoot Fe nutrition (S. Li *et al*., 2023). In maize, hemicellulose‐bound Fe contributes to tolerance against Fe‐deficiency‐induced chlorosis, with P‐dependent Fe inactivation in the root apoplast serving as a key regulatory factor (Shi *et al*., 2018).

Structural modifications also regulate Fe mobilization from the apoplast. Mutations in the *Cdi* gene, which alter the structure of rhamnogalacturonan‐II (RG‐II: complex polysaccharide component of pectin) in the cell wall, lead to impaired Fe reallocation, disrupted cell wall architecture, and stunted root growth under Fe deficiency (Peng *et al*., 2021). Exogenous abscisic acid (ABA) promotes Fe release from the apoplast by enhancing phenolic secretion, facilitating Fe mobilization from the apoplast (Lei *et al*., 2014). Collectively, these findings highlight the intricate interplay between cell wall modifications, nutrient interactions, and hormonal signaling in regulating apoplastic Fe redistribution, underscoring their critical roles in plant adaptation to Fe‐deficient conditions.

## Iron and cell wall acidification/expansion

**Fig. 3:**
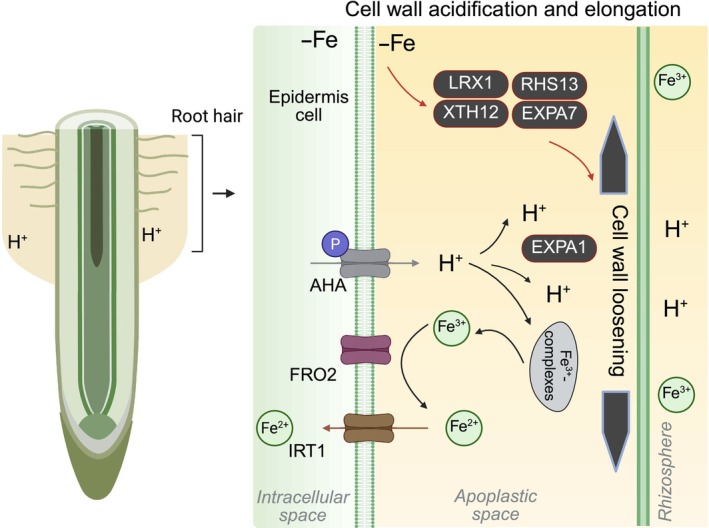
Impact of cell wall acidification and expansion on iron (Fe) uptake in roots. The plasma membrane H^+^‐ATPase (AHA) drives proton (H^+^) extrusion into the apoplast, establishing charge and pH gradients that are essential for both iron uptake and cell expansion. AHA activity acidifies the rhizosphere, increasing Fe^3+^ solubility and promoting its reduction to Fe^2+^ by ferric reduction oxidase 2 (FRO2), after which Fe^2+^ is imported into root cells via iron‐regulated transporter 1 (IRT1). AHA‐mediated acidification also activates expansin 1 (EXPA1), which loosens the cell wall, facilitating cell elongation and promoting root and root hair extension. Under iron deficiency, multiple proteins and genes involved in root hair elongation are upregulated, increasing root surface area and potentially enhancing Fe absorption. Examples include extracellular leucine‐rich repeat extensin protein (LRX1), xyloglucan endotransglucosylase 12 (XTH12), root hair specific 13 (RHS13), and expansin 7 (EXPA7). Black arrows indicate regulatory relationships between cell wall acidification and iron uptake, while red arrows indicate links between iron deficiency and cell expansion. Figure created with BioRender.com (https://BioRender.com/qsxvu5i).

The secretion of protons into the root apoplast generates charge and pH gradients that facilitate iron uptake, primarily in root hairs (Hoffmann *et al*., 2019). It drives secondary transporters necessary for nutrient absorption by modulating apoplastic pH (Fig. 3). Cell wall acidification involves the efflux of protons into the apoplast through the activation of plasma membrane (PM)‐localized P‐type H^+^‐ATPases (AHAs). The activity and localization on the PM of AHAs in Arabidopsis are crucial for plant growth and adaptation to various environmental conditions (Haruta *et al*., 2018). The H^+^‐ATPase‐mediated proton extrusion across the PM of rhizodermic cells is inducible by Fe deficiency, aiding in mobilizing sparingly soluble Fe sources. In Arabidopsis, PM AHAs are encoded by 11 AHA genes, among which AHA2 plays a key role in rhizosphere acidification through H^+^ extrusion in roots upon iron deficiency (Santi & Schmidt, 2009). AHA‐mediated acidification increases Fe^3+^ solubility, with a 1000‐fold increase in solubility for each unit drop in pH. During Fe deficiency, PDR9 (pleiotropic drug resistance 9) releases coumarins that facilitate Fe mobilization (Fourcroy *et al*., 2014). This process, although not directly linked to proton extrusion, represents an additional cell‐wall‐associated strategy contributing to rhizosphere Fe solubilization. These processes increase the availability of iron to plants by regulating the activity of iron‐regulated proteins/transporters at the membrane. FERRIC REDUCTION OXIDASE 2 (FRO2) catalyzes the reduction of Fe^3+^ to Fe^2+^ at the PM, thereby enabling subsequent uptake. Transporters such as IRON‐REGULATED TRANSPORTER 1 (IRT1) mediate the import of Fe^2+^ into the symplast (Brumbarova *et al*., 2015). IRT1, FRO2, and AHA2 interact dynamically to control high‐affinity Fe uptake in root epidermal cells of Arabidopsis (Martín‐Barranco *et al*., 2020). In citrus, AHA6 is required for rhizosphere acidification and Fe uptake, with its expression being transcriptionally regulated by MYB308 (Fan *et al*., 2022).

The chemistry of the cell wall is strongly affected by extracellular pH, which plays a crucial role in modulating cell wall elasticity and expansion (Xu & Yu, 2023). The acidification of plant primary cell walls by the H^+^‐ATPase is crucial for primary root growth (Miao *et al*., 2021). Auxin‐induced growth is characterized by PM H^+^‐ATPase‐mediated acidification of the cell wall, which facilitates cellular expansion and elongation (Hager, 2003; Barbez *et al*., 2017). In an acidic environment, wall‐loosening proteins become activated, which loosen the connections between different cell wall polysaccharides, leading to cell wall enlargement (Phyo *et al*., 2019). Expansins (EXP), a family of nonenzymatic proteins, play a central role in this process by mediating pH‐dependent cell wall loosening (Cosgrove, 2000; Samalova *et al*., 2023). In the root transition zone, AHA‐induced acidification facilitates EXPA1‐driven cell wall loosening, promoting cell elongation (Pacifici *et al*., 2018). Conversely, the intracellular accumulation of AHA2 correlates with reduced H^+^ secretion near the transition zone, thereby suppressing root growth (Haruta *et al*., 2018).

In response to iron deficiency, plants can increase root surface area by enhancing the development of root hairs and/or promoting root elongation (Santi & Schmidt, 2009; Gratz *et al*., 2019). Root hairs are long, thin extensions of root epidermal cells that play a crucial role in nutrient uptake. Specifically, in response to Fe deficiency, plants enhance root surface area by increasing root hair development (Santi & Schmidt, 2009). AHA7 activity has been identified as crucial for Fe‐deficiency‐induced root hair formation. In *aha7* mutants, the number of root hairs is significantly reduced under both normal and Fe‐deficient conditions (Santi & Schmidt, 2009). Iron deficiency induces the expression of numerous genes associated with cell wall remodeling and elongation, processes that are essential for proper root hair development and adaptive root architecture (Schwarz & Bauer, 2020). Examples include extracellular leucine‐rich repeat extensin protein (LRX1) (Baumberger *et al*., 2001); xyloglucan endotransglucosylase 12 (XTH12) (Haghir *et al*., 2025); root hair specific 13 (RHS13) (Tanaka *et al*., 2017); EXPA7 (Lin *et al*., 2011). In conclusion, surface pH is not only important for iron uptake but also modulates the activation of expansins and other cell wall‐modifying enzymes, which contribute to loosening the cell wall and facilitating root hair elongation (Hoffmann *et al*., 2019) (Fig. 3). These responses highlight the interplay between iron availability, cell wall remodeling, and environmental factors in determining root and root hair development and function.

## Iron and endodermal cell wall remodeling

**Fig. 4:**
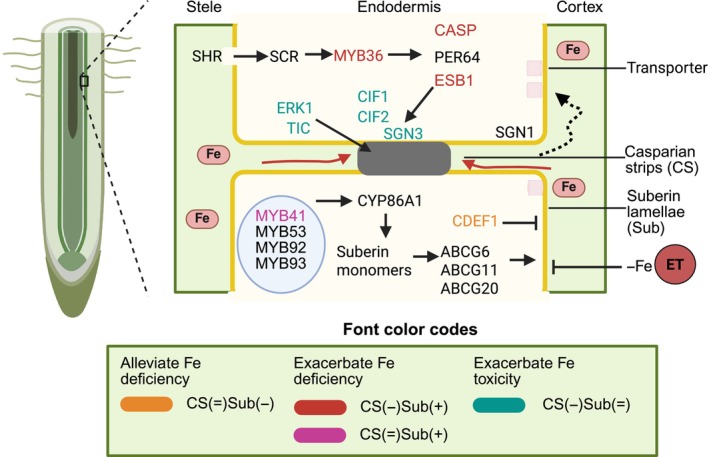
Schematic representation of the regulation of endodermal cell wall remodeling and iron (Fe) homeostasis in plants. The endodermis undergoes two major cell wall modifications: the formation of Casparian strips (CS; gray) and deposition of suberin lamellae (Sub; yellow), which together control endodermal permeability. The CS restricts passive solute movement (red arrows) and facilitates selective iron transport, while suberin lamellae further modulate this process (dotted black arrows). Multiple genes regulate the formation and maintenance of CS and suberin lamellae; changes in their expression affect the integrity of these barriers, thereby influencing Fe homeostasis. The interplay between suberization, CS formation, and Fe regulation is indicated by distinct font colors, as explained in the key below. Fe deficiency suppresses suberization through ethylene (ET) signaling (Barberon *et al*., 2016). Black arrows and inhibition symbols represent regulatory relationships. Figure created with BioRender.com (https://BioRender.com/taacu4u).

Nutrients move through the cell wall continuum of epidermal and cortical cells before reaching the endodermis, which regulates selective water and nutrient transport into vascular tissues (Li *et al*., 2018a). The endodermis serves as a critical checkpoint, where selective transport is mediated by two key barriers: the lignin‐based CS and the suberin lamellae (Figs 1, 4). During endodermal differentiation in Arabidopsis, these barriers form sequentially, with the CS limiting apoplastic diffusion and the suberin lamellae reinforcing selective transport (Enstone *et al*., 2002). Both CS and the suberin lamellae develop progressively from differentiated endodermal cells and are absent in the meristem and transition zones.

## The Casparian strip

The CS is a lignin‐rich, ring‐like structure in the primary cell walls of root endodermal cells, acting as a crucial diffusion barrier (Naseer *et al*., 2012). By restricting passive water and solute flow, the CS ensures selective nutrient uptake by compelling nutrients to transition from the apoplast into the symplast for regulated absorption (Barberon, 2017; Nakamura & Grebe, 2018; Li *et al*., 2018b). The formation of the CS in endodermal cells involves the precise spatial and temporal deposition of lignin, orchestrated by a complex regulatory network (Lee *et al*., 2013). Key transcription factors, such as SHORT‐ROOT (SHR) and SCARECROW (SCR), establish a hierarchical cascade that governs CS formation. SHR, expressed in the stele, is transported to endodermal cells via the symplastic pathway, where it regulates SCR. SCR, in turn, activates the expression of MYB36, a master regulator of endodermal differentiation (Kamiya *et al*., 2015). MYB36 directly induces genes critical for lignin polymerization, including CASPs (Casparian strip membrane domain proteins), PER64, and ENHANCED SUBERIN 1 (ESB1) (Li *et al*., 2018b). CASPs play a pivotal role by directing lignin deposition to specific PM sites, ensuring the localized assembly of the CS (Roppolo *et al*., 2011). This process is modulated by receptor‐like kinases, such as SCHENGEN1 (SGN1) and SCHENGEN3 (SGN3), which coordinate with ESB1 to reinforce CS integrity (Pfister *et al*., 2014; Alassimone *et al*., 2016). Peptide ligands, Casparian strip integrity factor 1 (CIF1) and CIF2, interact with their receptor SCHENGEN3 (SGN3) to play a crucial role in regulating CS formation (Nakayama *et al*., 2017).

The CS is a critical checkpoint for ion homeostasis and nutrient balance in plants. Its role in regulating iron translocation and maintaining nutrient equilibrium has been demonstrated in several studies. For example, *cif1 cif2* mutants exhibit discontinuous CS formation and sensitivity to excess iron, emphasizing the role of CS in adapting to fluctuating nutrient levels (Nakayama *et al*., 2017). For instance, mutations in *esb1* and *CASP1*/*CASP3* alter the shoot ionome, resulting in less iron accumulation compared with wild‐type plants (Hosmani *et al*., 2013). Similarly, *myb36* mutants, which regulate the expression of *CASP1*, *ESB1*, and *PER64*, exhibit aberrant leaf ionomes characterized by higher sodium (Na), magnesium (Mg), and zinc (Zn) levels but lower calcium (Ca), manganese (Mn), Fe, and boron (B) levels than the wild‐type (Kamiya *et al*., 2015). ENDODERMIS‐SPECIFIC RECEPTOR‐LIKE KINASE 1 (ERK1), along with ROP BINDING KINASE 1 (RBK1) to a lesser extent, is essential for CS formation, lignin deposition, and CASP1 localization (Durr *et al*., 2021). Mutants lacking *ERK1* and *RBK1* show increased root iron accumulation compared with wild‐type plants. ERK1 works with the circadian regulator TIME FOR COFFEE (TIC) in a signaling pathway crucial for CS organization. Under excessive iron conditions, ERK1 expression increases in a pH‐dependent manner. Mutants lacking ERK1 (*erk1‐3*) or TIC (*tic‐2*) show heightened sensitivity to high iron levels, altered root microbiomes, and increased root iron accumulation, highlighting the importance of intact endodermal barriers in maintaining iron homeostasis (Durr *et al*., 2021). The role of CS in nutrient regulation extends to other species. In soybean (*Glycine max*), signaling associated with iron deficiency chlorosis indicates that many genes involved in CS formation are upregulated under iron deficiency (Moran Lauter *et al*., 2014). Further investigations in rice (*Oryza sativa*) provide additional insights into the role of CS in nutrient regulation. The *Oscasp1‐3* mutant, for example, exhibits defective ion homeostasis, with significantly higher concentrations of iron, manganese, and sodium but lower concentrations of potassium and arsenic under nutrient stress (Yang *et al*., 2022). In conclusion, CS plays an important role in iron and other nutrient regulation in plant roots, integrating structural and molecular mechanisms to ensure selective transport, maintain nutrient balance, and support environmental adaptability.

## Suberization

Suberin lamellae, a hydrophobic secondary cell wall layer around endodermal cells, are essential for root function and plant adaptation (Barberon, 2017; Shukla & Barberon, 2021). This barrier develops progressively, with patchy deposition near the root tip and continuous coverage in mature zones, except for nonsuberized passage cells near xylem poles (Barberon *et al*., 2016). Suberin, a complex heteropolymer, consists of aliphatic polyesters and phenolic compounds, synthesized via distinct pathways and transported to the cell wall by ABC transporters like ABCG6, ABCG11, and ABCG20 (Landgraf *et al*., 2014; Barberon, 2017). Enhanced suberization can compensate for defects in the CS, demonstrating redundancy in mature endodermal cells.

Mutants exhibiting alterations in the CS and ectopic suberin formation, such as *myb36*, *esb1*, *casp1*, and *casp3*, display reduced iron and manganese levels in leaves (Hosmani *et al*., 2013; Kamiya *et al*., 2015). The MYB transcription factors MYB41, MYB53, MYB92, and MYB93 are regulators in the primary response to suberin‐inducing signals. The transgenic line expressing MYB41 under the control of the ENDODERMAL LIPID TRANSFER PROTEIN promoter (ELTP::MYB41) exhibits constitutively enhanced suberization without defects in the CS. Notably, this line also shows reduced iron accumulation levels in leaves (Shukla *et al*., 2021). The *sgn3‐3* mutant, which has an altered CS but unaffected suberin content, exhibits high sensitivity to elevated iron conditions (Durr *et al*., 2021). The cytochrome P450 fatty acid omega‐hydroxylase CYP86A1 is a crucial enzyme in aliphatic root suberin biosynthesis in Arabidopsis. The *cyp86a1* mutant exhibits reduced or altered suberin (Höfer *et al*., 2008). Notably, mutations in this gene in poplar roots lead to disrupted suberin lamella deposition, significantly decrease Fe content in leaves, and strongly induce the iron deficiency response in roots (Grünhofer *et al*., 2024). Cutinase CDEF1 (CUTICLE DESTRUCTING FACTOR1) functions in suberin degradation. The line, in which CDEF1 is expressed under the control of the ELTP promoter, ELTP::CDEF1, also exhibits reduced suberin content while maintaining a functional CS (Naseer *et al*., 2012). Expressing ELTP::CDEF1 in *irt1* mutants reduces suberization, rescuing the growth of the *irt1* mutant (Barberon *et al*., 2016). The interplay between suberization and CS formation underscores a dynamic system that optimizes iron acquisition (Fig. 4).

Nutrient deficiencies exert differential effects on suberization. Potassium (K) and sulfur (S) deficiencies promote suberization through ABA signaling, while deficiencies in Fe, zinc (Zn), and manganese (Mn) suppress it via ethylene signaling (Barberon *et al*., 2016). The antagonistic crosstalk between ABA and ethylene fine‐tunes suberization. Mutants deficient in ABA biosynthesis (*aba2*) or signaling (*abi3*, *abi4*, *abi5*) exhibit delayed and discontinuous suberization, underscoring ABA's critical role (Barberon *et al*., 2016). Conversely, iron deficiency reduces suberization, enhancing endodermal permeability to facilitate iron uptake (Fig. 4). Mutants such as *irt1* and *nramp1* display reduced suberization, mirroring responses to Fe and Mn deficiencies (Barberon *et al*., 2016). The plasticity of suberization is pivotal for ion homeostasis and stress resilience. In iron‐deficient conditions, suberin‐specific peroxidase activity decreases, reflecting the nuanced regulation of suberin deposition (Sijmons *et al*., 1985).

Gene regulatory networks in rice reveal the developmental plasticity of the root exodermis in response to water deficit, upregulating suberin biosynthesis while downregulating Fe ion transport (Reynoso *et al*., 2022). These networks orchestrate structural and physiological adaptations to optimize nutrient uptake and stress resilience. GULONO‐1,4‐Γ‐LACTONE OXIDASE 2 (GULLO2) has been implicated in facilitating Fe transport from the endosperm into developing embryos in Arabidopsis. This process is closely linked to the regulation of seed coat suberization (Murgia *et al*., 2023). Suberin's plasticity and redundancy are essential for maintaining ion homeostasis and protecting against environmental challenges. This adaptability enables plants to modulate endodermal differentiation to balance nutrient uptake and defense mechanisms under fluctuating conditions.

## Iron and callose deposition on the cell wall

Callose, a β‐1,3‐glucan polysaccharide, is mainly produced in special cell walls and exercises important functions. It is commonly found in pollen tubes, cell plates of dividing cells, and cells affected by wounding or fungal infection (Amsbury *et al*., 2018; N. Li *et al*., 2023; Hsieh *et al*., 2024). Plasmodesmata (PD), which connect the cytoplasm of neighboring cells, are crucial for symplastic molecular exchanges (Yan & Liu, 2020). The synthesis or degradation of callose near the neck zone of PD regulates their permeability and transport capacity (Vatén *et al*., 2011; Zavaliev *et al*., 2011; Wu *et al*., 2018). This permeability is influenced by numerous developmental processes and environmental stresses, which modulate cell‐to‐cell molecular trafficking and signaling (Burch‐Smith & Zambryski, 2012; Liu *et al*., 2022). There is increasing evidence that ROS and redox signaling regulate callose deposition and symplastic permeability (Stonebloom *et al*., 2009; Benitez‐Alfonso *et al*., 2011).

**Fig. 5:**
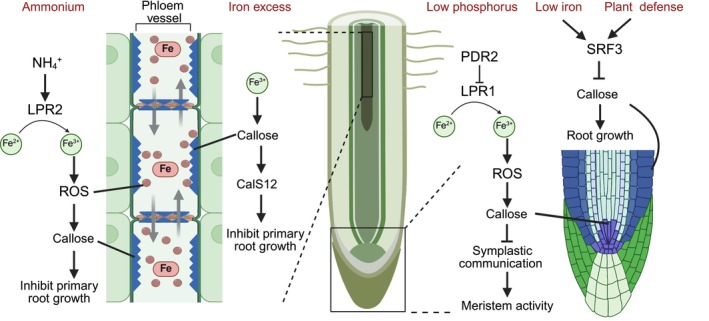
Schematic representation of how iron (Fe) availability regulates callose deposition and impacts root physiology. Ammonium (NH_4_^+^) stress and excess Fe trigger alterations in callose deposition within the phloem, leading to reduced phloem transport and inhibition of root growth. In the root apical meristem (RAM), Fe‐dependent reactive oxygen species (ROS) production under low phosphate (Pi) conditions promotes callose synthesis, which decreases plasmodesmata (PD) permeability, thereby impairing stem cell maintenance and RAM activity. In addition, STRUBBELIG receptor kinase 3 (SRF3) acts as a sensor of Fe deficiency to fine‐tune callose deposition, ensuring appropriate nutrient flux and maintaining immune preparedness. Black arrows and inhibition symbols represent regulatory relationships. LPR1/2, low phosphate root 1/2; PDR2, phosphate deficiency response 2. Figure created with BioRender.com (https://BioRender.com/lxv4xgw).

The phloem distributes photosynthetic products for metabolism and storage across long distances, relying on specialized enucleated sieve elements interconnected by sieve pores that enable efficient sap movement (Kalmbach *et al*., 2023). In roots, callose deposition within phloem cells is vital for maintaining phloem functionality and protecting the vascular system under stress conditions (Barratt *et al*., 2011; Xie *et al*., 2011). Recent findings suggest that ammonium (NH_4_^+^) stress triggers pronounced callose deposition in the phloem through LPR2‐mediated Fe overaccumulation in the apoplast. This aberrant iron accumulation triggers a burst of ROS, impairing phloem function (Reyt *et al*., 2015). Thus, ROS acts downstream of iron deposition to induce callose deposition in response to NH_4_^+^ stress (Liu *et al*., 2022). Excess iron can also lead to callose deposition in the phloem. Research has shown that high iron levels inhibit primary root growth and decrease PD permeability, with callose deposition in the phloem being a significant factor (O'Lexy *et al*., 2018). Genetic mutant screening identified GSL5/CalS12 enzymes as responsible for the iron response (O'Lexy *et al*., 2018). In conclusion, environmental cues, such as NH_4_^+^ stress and excess iron, lead to changes in callose deposition in the phloem (Fig. 5). These responses are governed by intricate crosstalk between iron accumulation, ROS signaling pathways, and the activity of specific genetic regulators.

In root development, PD and callose turnover are essential for the movement of the SHORT‐ROOT (SHR) protein (Vatén *et al*., 2011) and for determining lateral root formation patterns (Benitez‐Alfonso *et al*., 2013). However, the mechanisms linking callose‐regulated cell‐to‐cell signaling in the root apical meristem (RAM) to soil‐borne cues are not well understood. Phosphorus (P) and Fe are known to influence root length. The low phosphate effects on the root meristem are mitigated by removing iron from the media (Ward *et al*., 2008). In Arabidopsis, Pi (inorganic phosphate) deficiency inhibits RAM activity due to increased Fe bioavailability and its associated toxicity, with root responses (callose deposition, reduced growth, and stem cell population depletion) being attributed to iron toxicity (Müller *et al*., 2015). Iron also plays a key role in remodeling primary root development in response to P deficiency, mediated by LOW PHOSPHATE ROOT 1 (LPR1). LPR1, a ferroxidase, catalyzes the oxidation of Fe^2+^ to Fe^3+^, and under low P conditions, its activity leads to ROS generation, inducing callose synthesis and deposition at PD zones in root cells (Müller *et al*., 2015). This process impairs symplastic communication, as shown by reduced SHR movement, leading to diminished stem cell maintenance and RAM activity. The PHOSPHATE DEFICIENCY RESPONSE 2 (PDR2)–LPR1 module mediates Fe accumulation and ROS generation, causing callose and pectin deposition in the root's meristem and elongation zones (Müller *et al*., 2015). This restricts SHR movement and interferes with root meristem differentiation, with the CLAVATA3/ENDOSPERM SURROUNDING REGION (CLE14) signaling pathway being involved in RAM differentiation in response to iron mobilization under low Pi conditions. In rice, low phosphorus induces iron and callose homeostatic regulation, with Fe playing a minor role in root morphological remodeling under low P (Ding *et al*., 2018). In conclusion, root development under low phosphorus conditions is intricately regulated by iron‐dependent callose deposition and ROS generation, which impair symplastic signaling and root meristem activity (Fig. 5).

Callose deposition is a well‐established marker of plant immunity (Luna *et al*., 2011). Previously, it was reported that iron deficiency not only reduced callose deposition but also exhibited reduced symptom severity in Arabidopsis challenged with the phytopathogen, an enterobacterium *Dickeya dadantii*, and the necrotrophic fungus *Botrytis cinerea* (Kieu *et al*., 2012). Recent findings highlight the role of STRUBBELIG RECEPTOR KINASE 3 (SRF3), a receptor kinase sensing low iron levels, in mediating callose deposition and maintaining signaling for growth and iron homeostasis in root tips (Platre *et al*., 2022). SRF3 balances nutrient availability and defense by fine‐tuning iron homeostasis and activating immune response genes without disrupting cell‐to‐cell movement. This mechanism ensures continued nutrient flow and signaling, crucial for root development under fluctuating environmental conditions. By integrating nutrient sensing and immune priming, SRF3 reinforces the plant's ability to adapt to low‐iron conditions while preserving developmental integrity (Platre *et al*., 2022). In conclusion, there might be a link between iron availability and callose deposition during plant defense and microbial pathogenicity (Fig. 5).

## Conclusion and future directions

Emerging evidence highlights the root cell wall as a dynamic, actively remodeled interface that governs Fe uptake and distribution. In response to fluctuating Fe availability and environmental stresses, root cell walls undergo structural and biochemical changes that influence nutrient transport and storage. Integrating physiological, structural, and molecular perspectives, we propose the concept of ‘root cell wall plasticity’ in Fe regulation, an approach that opens new avenues in plant nutrition research. Future research should elucidate the signaling networks, hormonal pathways, and regulatory mechanisms that connect ROS production, lignin biosynthesis, pectin modification, and cell wall loosening enzymes in Fe‐responsive remodeling, providing a timely framework to leverage advances in imaging, single‐cell transcriptomics, and genome editing.

Soil composition profoundly shapes root cell wall plasticity and Fe acquisition, but the mechanisms linking these processes under field conditions remain largely unexplored. Fe solubility is strongly affected by soil pH: it is poorly available in alkaline, calcareous soils (high pH, high CaCO_3_) but becomes highly soluble and potentially toxic in strongly acidic soils. High pH and excessive calcium can physically modify root cell walls, whereas in Fe‐rich acidic environments, plants frequently reinforce apoplastic barriers to limit Fe uptake. Elucidating how these cell wall modifications respond to soil pH across contrasting alkaline and acidic conditions would substantially advance our understanding of Fe homeostasis. The rhizosphere microbiome further modulates cell wall plasticity. Plant growth–promoting rhizobacteria and fungi influence phytohormone levels and trigger defense‐related responses, which in turn modify cell wall structure and Fe availability at the root–soil interface. This plant–microbe–cell wall interplay adds a layer of ecological complexity to Fe regulation. Moreover, abiotic stresses such as drought and salinity induce lignification and suberization, reinforcing apoplastic barriers that restrict uncontrolled water and ion flux. While these adjustments enhance stress tolerance, they can concurrently affect Fe acquisition. Deciphering the mechanisms that integrate these factors will not only advance fundamental plant biology but also inform strategies to optimize Fe nutrition and enhance stress resilience in crops grown under diverse soil conditions.

Overall, addressing the outstanding questions outlined in Box 1 will be critical for understanding how plants maintain Fe balance under changing environmental conditions and for translating this knowledge into resilient, nutrient‐efficient crop varieties.

Box 1 **Outstanding questions**

How do plants coordinate cell wall modifications with molecular Fe sensing and signaling pathways?

What triggers specific responses like lignification or callose deposition under Fe toxicity?

How is apoplastic Fe mobilized and reallocated during deficiency, and how does this interplay with symplastic and transcellular transport?

What are the precise roles of ROS in mediating the crosstalk between Fe stress and cell wall dynamics?

How do these diverse mechanisms integrate spatially and temporally within different root tissues to optimize Fe uptake, distribution, and storage?

## Competing interests

None declared.

## Author contributions

PK conceptualized the review, wrote the original draft, prepared the figures, reviewed and edited the article. PB reviewed the article and acquired funding.

## Disclaimer

The New Phytologist Foundation remains neutral with regard to jurisdictional claims in maps and in any institutional affiliations.
